# Immunization With the CSF-470 Vaccine Plus BCG and rhGM-CSF Induced in a Cutaneous Melanoma Patient a TCRβ Repertoire Found at Vaccination Site and Tumor Infiltrating Lymphocytes That Persisted in Blood

**DOI:** 10.3389/fimmu.2019.02213

**Published:** 2019-09-18

**Authors:** Mariana Aris, Alicia Inés Bravo, Heli Magalí Garcia Alvarez, Ibel Carri, Enrique Podaza, Paula Alejandra Blanco, Cecilia Rotondaro, Sofia Bentivegna, Morten Nielsen, María Marcela Barrio, José Mordoh

**Affiliations:** ^1^Centro de Investigaciones Oncológicas-Fundación Cáncer, Buenos Aires, Argentina; ^2^Unidad de Inmunopatología, Hospital Interzonal General de Agudos Eva Perón, Buenos Aires, Argentina; ^3^IIBIO-UNSAM, Buenos Aires, Argentina; ^4^Fundación Instituto Leloir, IIBBA-CONICET, Buenos Aires, Argentina; ^5^Department of Health Technology, Technical University of Denmark, Lyngby, Denmark; ^6^Instituto Alexander Fleming, Buenos Aires, Argentina

**Keywords:** cutaneous melanoma, CSF-470 vaccine, vaccination site, cutaneous metastasis, tumor infiltrating lymphocytes, TCRβ repertoire

## Abstract

The CSF-470 cellular vaccine plus BCG and rhGM-CSF increased distant metastases-free survival in cutaneous melanoma patients stages IIB-IIC-III relative to medium dose IFN-α2b (CASVAC-0401 study). Patient-045 developed a mature vaccination site (VAC-SITE) and a regional cutaneous metastasis (C-MTS), which were excised during the protocol, remaining disease-free 36 months from vaccination start. CDR3-TCRβ repertoire sequencing in PBMC and tissue samples, along with skin-DTH score and IFN-γ ELISPOT assay, were performed to analyze the T-cell immune response dynamics throughout the immunization protocol. Histopathological analysis of the VAC-SITE revealed a highly-inflamed granulomatous structure encircled by CD11c^+^ nested-clusters, brisk CD8^+^ and scarce FOXP3^+^, lymphocytes with numerous Langhans multinucleated-giant-cells and macrophages. A large tumor-regression area fulfilled the C-MTS with brisk lymphocyte infiltration, mainly composed of CD8^+^PD1^+^ T-cells, CD20^+^ B-cells, and scarce FOXP3^+^ cells. Increasing DTH score and IFN-γ ELISPOT assay signal against the CSF-470 vaccine-lysate was evidenced throughout immunization. TCRβ repertoire analysis revealed for the first time the presence of common clonotypes between a VAC-SITE and a C-MTS; most of them persisted in blood by the end of the immunization protocol. *In vitro* boost with vaccine-lysate revealed the expansion of persistent clones that infiltrated the VAC-SITE and/or the C-MTS; other persistent clones expanded in the patient's blood as well. We propose that expansion of such persistent clonotypes might derive from two different although complementary mechanisms: the proliferation of specific clones as well as the expansion of redundant clones, which increased the number of nucleotide rearrangements per clonotype, suggesting a functional antigenic selection. In this patient, immunization with the CSF-470 vaccine plus BCG and rhGM-CSF induced a T-cell repertoire at the VAC-SITE that was able to infiltrate an emerging C-MTS, which resulted in the expansion of a T-cell repertoire that persisted in blood by the end of the 2-year treatment.

## Introduction

Treatment of cutaneous melanoma (CM) has greatly improved in recent decades due to two main discoveries. First, it was found that tumors from about half of CM patients carry the driver activating mutation V600E in the BRAF oncogene (1), for which inhibitors such as vemurafenib (2), and dabrafenib (3) have been designed. The use of such inhibitors, to which MEK inhibitors, such as cobimetinib and trametinib, were later added, produced good clinical results in stage-IV CM patients (4, 5) and more recently in adjuvancy in stage-III CM patients (6). However, the common development of drug resistance is the main drawback of these treatments (7).

Second, different lines of research led to the discovery of immune checkpoints, such as the CTLA-4/CD80-86 (8) and PD1/PD-L1 (9) axes, whose roles are to turn-off antigen(Ag)-stimulated lymphocytes, presumably to prevent over-activity. Blocking those regulatory axes with Immune Checkpoint Inhibitory (ICKI) monoclonal antibodies (MAbs) produced impressive clinical results, both in advanced CM (10) and in non-small cell lung carcinoma (11). Recently, the anti-PD1 pembrolizumab has been reported to be also effective as adjuvant treatment in stage-III CM patients (12). The rationale behind this clinical effect is that ICKI relieve CD8^+^ lymphocytes from exhaustion, and since they are administrated without any previous immune stimulation, it is assumed that anti-tumor reactive lymphocytes already exist in cancer patients in number enough so that previous stimulation is unnecessary. However, evidence has been found that the clinical result of ICKI is better in the measure that tumors are more heavily infiltrated by lymphocytes; therefore, an increase in tumor-reactive lymphocytes previous to ICKI administration would be beneficial (13).

We have demonstrated in a randomized Phase II adjuvant study (CASVAC-0401, NCT01729663), that the CSF-470 cellular vaccine in combination with BCG and rhGM-CSF, increased distant metastases-free survival in stages IIB, IIC, and III CM patients with respect to medium dose IFN-α2b (14). In the same study, it was demonstrated that T-cell reactivity against vaccine Ags was maintained over the 2 years (2-yr) treatment without signs of exhaustion. Besides, only low numbers of T lymphocytes reactive to melanoma cells were detected in blood before treatment, whereas after vaccination they strongly increased (15). In a previous analysis of vaccinated patient-006 from the CASVAC-0401 trial, an increase in tumor infiltrating lymphocytes (TIL) in a cutaneous metastasis (C-MTS) developing at the end of the immunization protocol was observed. This lesion was infiltrated with a T-cell repertoire that expanded in blood over the 2-yr treatment, persisting 2-yr after completing the protocol; along with lymphocytes only detected at the tumor site (16). In this paper, we investigated in-depth another patient from the same protocol; we were able to compare for the first time clonotypes from a vaccination site (VAC-SITE), a C-MTS, and peripheral blood throughout the CSF-470 immunization protocol.

## Case Report

### Patient's 045 Case Presentation and Treatment

Patient #045 (pt-045) is a 51 year-old Caucasian man, to whom in January 2016 a primary dorsal epithelioid CM (1 CM) was excised. Its main characteristics were a 2.3 mm Breslow thickness, an intermediate proliferative index (23% Ki-67^+^), and non-brisk lymphoid infiltration; no evidence of ulceration, regression, satellitosis, or vascular invasion were found (Supplementary Figure 1). One month later, a right axillary sentinel lymph node (LN) was excised, and a micrometastasis was found. Pt-045 was therefore at stage-IIIA (AJCC 8th edition), but radical lymphadenectomy was not performed. In April 2016, the patient signed the informed consent and entered the CASVAC-0401 study, being assigned to the CSF-470 vaccine arm. In August 2016, after receiving four vaccinations, a CM recurrence on the scar (Scar-CM) was excised, which displayed scarce CD8^+^ lymphocyte infiltration (*not shown*). The patient continued treatment as *per* protocol. In May 2017, after 8 vaccinations and 12 months after starting treatment, the patient presented an enlarged right-axillary LN and a thoracic *in-transit* cutaneous metastasis (C-MTS). A radical axillary LN resection and a C-MTS was resected; 1/20 metastatic LN was found. In the same surgical procedure, three vaccination nodules (VAC-SITE), all metabolically active, were excised at the patient's decision. After surgery, pt-045 continued and completed the 2-yr immunization protocol with the CSF-470 vaccine without further events, and without evidence of disease 36 months from protocol start. In the present study, one VAC-SITE and the C-MTS were analyzed in detail. Time course of treatment, as well as the surgical events and blood extractions, are indicated (Supplementary Figure 2).

## Results

### Analysis of a CSF-470 VAC-SITE

**Figure 1:**
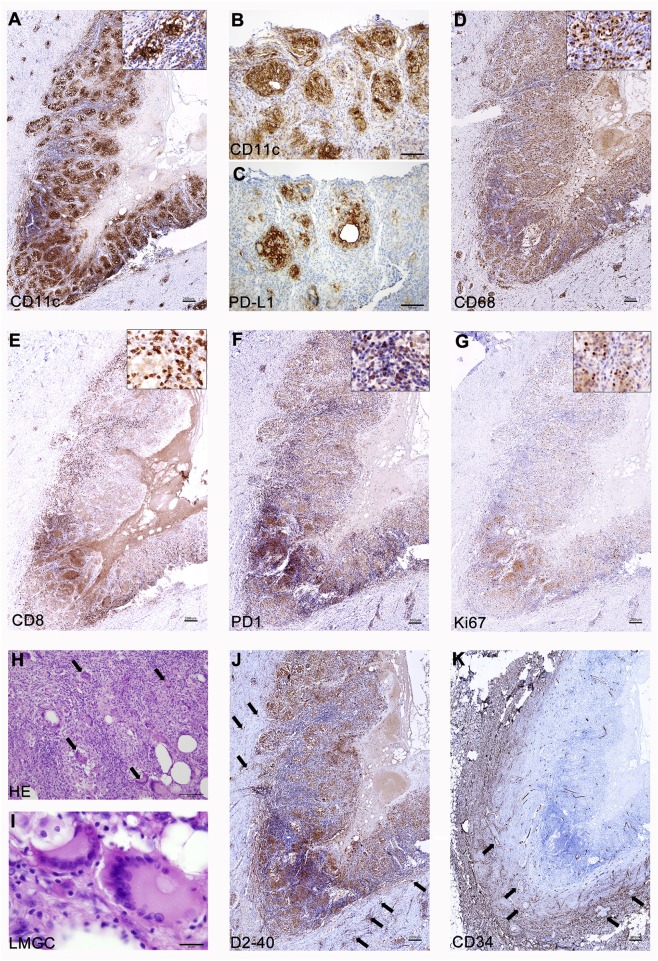
Analysis of a CSF-470 VAC-SITE tissue. **(A–C)** Dense clusters of CD11c^+^ cells were observed at the VAC-SITE, most of them also expressing PD-L1; **(D)** Abundant CD68^+^ macrophages were also present in the area. **(E–G)** CD8^+^ lymphocytes were mainly PD1^−^ and many of them were proliferating (Ki67^+^). **(H,I)** Numerous LMGC were observed by HE staining (arrows). **(J,K)** The VAC-SITE was surrounded by numerous lymphatic and blood vessels (arrows). Original magnifications = **(A,D–G,J,K)**: 20X; **(B,C,H)**: 100X; **(I)**: 1000X. Scale bars: **(A,D–G,J,K)** =200 μm; **(B,C,H)** = 100 μm; **(I)** = 20 μm.

One of the unanswered questions about repeated vaccinations with CSF-470 plus BCG and rhGM-CSF is the cellular composition of the VAC-SITE, since their systematic analysis was not contemplated *per* protocol. The three VAC-SITES excised from pt-045 presented similar histological characteristics; only one is described here in detail. A highly-inflamed granulomatous structure was observed, with a necrotic center bordered by CD11c^+^ clusters, many of them PD-L1^+^ (Figures 1A–C). Such aggregates were surrounded by mostly CD8^+^ PD1^−^ lymphocytes (Figures 1E,F), some of them Ki-67^+^ (Figure 1G). In contrast, FOXP3^+^ lymphocytes were scarce (*not shown*). Abundant and dispersed CD68^+^ macrophages were found (Figure 1D). Large numbers of Langhans multinucleated giant cells (LMGC) were observed, some of them with more than 10 nuclei, which showed a typical circular peripheral arrangement (Figures 1H,I); no BCG bacilli were observed within these cells or elsewhere (*not shown*). The site contained D2-40^+^ lymphatic vessels (Figure 1J); abundant angiogenesis was revealed by CD34^+^ blood vessels (Figure 1K).

### Lymphoid Infiltration in a Regional C-MTS

**Figure 2:**
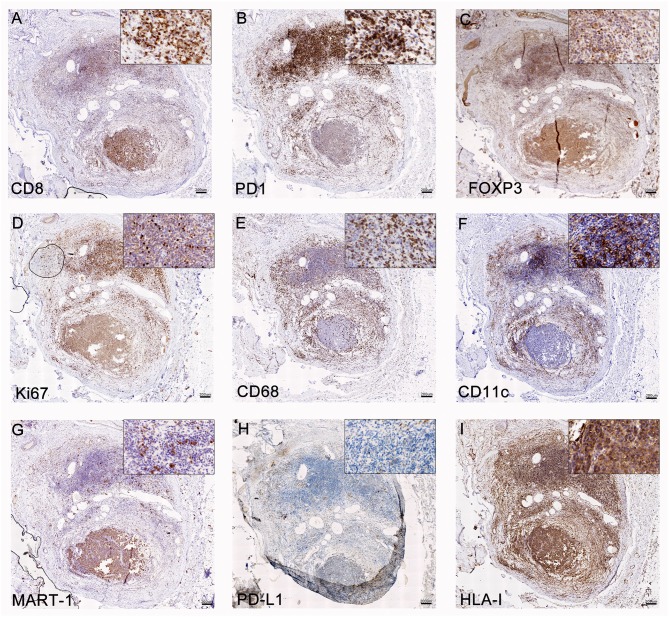
Analysis of C-MTS tissue. In the area of tumor regression (upper part of the biopsy), CD8^+^ **(A)** and PD-1^+^ **(B)** T lymphocytes were mainly present, while FOXP3^+^ Treg were scarce **(C)**; these lymphocytes were proliferating as determined by Ki-67^+^ staining **(D)**. Also, CD68^+^ macrophages **(E)** and CD11c Ag-presenting cells **(F)** were mainly concentrated in this area. In the lower part of the biopsy, MART-1^+^ **(G)** PD-L1^−^ **(H)** HLA class I ^+^ **(I)** viable tumor cells were observed. Original magnification = 20X. Scale bars = 200 μm.

From all the metastases that may signal the recurrence of melanoma, C-MTS are the most amenable to diagnostic biopsies. Besides, according to the CASVAC-0401 protocol, loco-regional metastases may be excised and patients still remain in the study. Analysis of C-MTS from pt-045 revealed tumor regression encompassing >90% of the nodule volume; the regression area was fulfilled with brisk lymphocyte infiltration, mainly composed of CD8^+^ T cells (Figure 2A), PD1^+^ T cells (Figure 2B), few FOXP3^+^ Treg cells (Figure 2C), and CD20^+^ B cells (*not shown*). Ki-67^+^ immune cells were identified in the infiltrated area (Figure 2D). Also, abundant CD68^+^ macrophages (Figure 2E) and dense infiltration of CD11c^+^ cells (Figure 2F) were detected. In the areas of brisk lymphocyte infiltration, scarce viable tumor cells were observed; Granzyme-B staining revealed positive cytosolic and nuclear granules in tumor cells in these areas of rich CD8^+^ lymphocytes infiltration (Supplementary Figure 3). A small portion of the cutaneous nodule contained MART-1^+^HLA-class I^+^PD-L1^−^ melanoma cells (Figures 2G–I), whereas few CD8^+^ PD1^+^ T cells attached to tumor cells were seen (Figures 2A,B). Abundant CD34^+^ blood vessels and D2-40^+^ lymphatic vessels were seen in the viable tumor area (*not shown*).

### Evidence of T-Cell Response Induced by the CSF-470 Vaccine

As a measure of the immune stimulation developed during CSF-470 vaccination, a skin DTH-score was performed at baseline (PRE) and after administration of each of the 13 vaccine doses; the score was calculated as described (14). As for most patients in the CASVAC-0401 study, pt-045 DTH-score increased with vaccination, evidencing the induction of a memory immune response to CSF-470 vaccine Ags (Supplementary Figure 4).

In order to characterize the repertoire of T-cell clones present at the VAC-SITE, recruited at the C-MTS, and their dynamics in blood during vaccination, CDR3-TCRβ sequencing from gDNA was performed in VAC-SITE, C-MTS as well as in PBMC samples obtained before (PBMC-PRE), during and at the end of the CSF-470 immunization (PBMC-POST-2 and PBMC-POST-3, respectively) (Supplementary Figure 2). Given the relative sample sizes used for the TCR sequencing, as expected, TCRβ repertoire was larger at PBMC samples than at VAC-SITE and C-MTS samples (Supplementary Figure 5A). Analysis of productive amino-acidic sequence clonotypes was performed (Supplementary Tables 1–5, Supplementary Material 1). Regarding general TCRβ metrics, the cumulative frequency of the 100 most-frequent peripheral TCRβ clonotypes (TOP100) was stable in time and amounted to around 10% of total clones; instead, TCRβ repertoires from the VAC-SITE and the C-MTS were more oligoclonal (Supplementary Figure 5B). Accordingly, plots of cumulative frequency of clones, ordered from highest to lowest, supported this distribution (Supplementary Figure 5C). Also, we analyzed in all samples the distribution of *redundant clones*, i.e., TCRβ clones with different gene rearrangements that converge into the same amino-acidic sequence; and their *specific redundancy*, the number of rearrangements *per* redundant clone. Both VAC-SITE and C-MTS presented a major cumulative frequency and mean proportion of redundant clones (Supplementary Figures 5D,E). Notably, TOP100 clones were enriched in redundant clones in every sample tested (Supplementary Figure 5F).

We addressed whether the T-cell repertoire found at the VAC-SITE was related to the C-MTS which appeared during the immunization protocol, as there might be clonotypes targeting shared-Ags. Indeed, 1,098 clones were found in common between the VAC-SITE and the C-MTS (Supplementary Figure 6A), which represented 37% of the total C-MTS (TIL) clones. Most of such shared clones were also detected in blood; only 14% were exclusively detected at tissue (*not shown*). Remarkably, the frequency of the shared clonotypes was significantly higher in C-MTS compared to VAC-SITE (Supplementary Figure 6B) adding together a cumulative frequency of 42%, suggesting their biological relevance (Supplementary Figure 6C).

**Figure 3:**
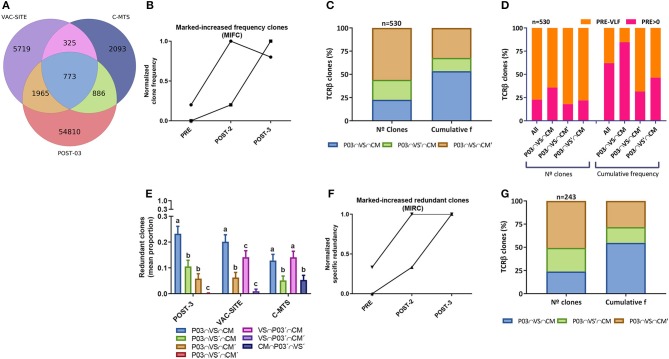
Analysis of *in vivo* persistent TCRβ clones following CSF-470 immunization. **(A)** Venn diagram showing the distribution of common TCRβ clonotypes between PBMC-POST-3, VAC-SITE, and C-MTS samples from pt-045. **(B)** Normalized frequency tracking of 2 different clonotypes to illustrate markedly-increased frequency clones, MIFC. **(C)** Distribution (%) of persistent MIFC regarding N° clones and cumulative frequency in the VAC-SITE/C-MTS compartments. **(D)** Distribution of MIFC and subsets regarding procedence from very-low-frequency clones (PRE-VLF) or basal pre-existing clones (PRE > 0). **(E)** Mean proportion of redundant clones for each subset within a sample (bootstrap *n* = 300, 100 iterations); bars with different letters are statistically different (Wilcoxon, *p* < 0.05). **(F)** Normalized specific redundancy tracking of 2 different clonotypes to illustrate normalized markedly-increased redundant clones, MIRC. **(G)** Distribution (%) of persistent MIFC regarding N° clones and cumulative frequency in the VAC-SITE and/or C-MTS compartments. *Samples:* PBMC-POST-3 = POST-3 = P03; VAC-SITE = VS; C-MTS = CM.

Another relevant question was whether the T-cell clones recruited at the VAC-SITE and/or the C-MTS persisted in blood by the end of the 2-yr immunization protocol (PBMC-POST-3), defined as *persistent clones*. Of the 1,098 T-cell clones common to the VAC-SITE and the C-MTS, 70% (773) were detected in the PBMC-POST-3 sample; 1,965 clones were only shared between the VAC-SITE and PBMC-POST-3; while 886 clones were only shared between the C-MTS and PBMC-POST-3; adding together 3,624 persistent clones (Figure 3A). Persistent clones made up 88% of PBMC-POST-3 TOP 100 most-frequent clones (*not shown*). Clone-tracking patterns were defined to study PBMC dynamics in blood throughout immunization, including persistent clones (Supplementary Figure 7). A subset of 530 persistent clones (27%) at least doubled their frequency throughout immunization, so called *markedly-increased frequency clones (MIFC)* (Figure 3B). Most circulating MIFC presented common clonotypes with the VAC-SITE, although the higher cumulative frequency with VAC-SITE/C-MTS common clones was found (Figure 3C). Regarding initial abundance, most MIFC expanded from very-low-frequency (VLF) clones; however there was a marked-expansion from *pre-existing basal* clones for VAC-SITE/C-MTS common clonotypes (Figure 3D). Regarding redundant clones, they were preferentially distributed among the persistent common clones between PBMC-POST-3 and VAC-SITE and/or C-MTS samples (Figure 3E). Almost all redundant clones varied their number of rearrangements in time (specific redundancy, *not shown*). Indeed, 243 of all persistent clones (13%) at least doubled their specific redundancy, so called *markedly-increased redundant clones (MIRC)* (Figure 3F). 56% of MIRC were also MIFC (*not shown*). Accordingly, MIRC presented more common clonotypes with the VAC-SITE, while the higher cumulative frequency with common VAC-SITE/C-MTS clones was found (Figure 3G).

### Analysis of PBMC Clonotypes Stimulated *in vitro* by the CSF-470 Vaccine

**Figure 4:**
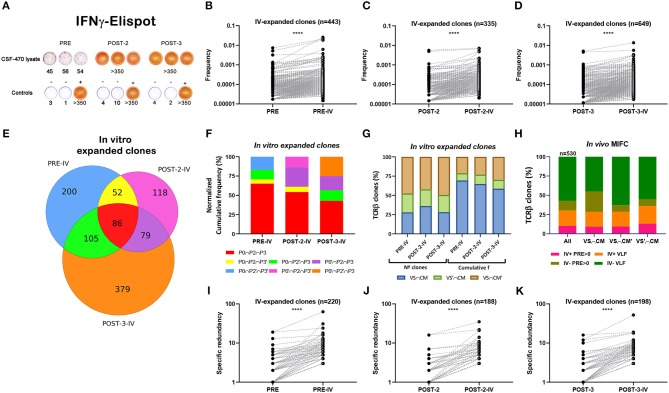
Analysis of persistent *in vitro* expanded TCRβ clones with CSF-470 vaccine Ags. **(A)** PBMC were stimulated *in vitro* (***iv***) with CSF-470-vaccine lysate and evaluated IFN-γ release by ELISPOT assay. Controls: PBMC with only culture medium (–), and with OKT3 plus PHA (+). **(B–D)** Paired-frequency of persistent *iv-*expanded TCRβ clonotypes relative to their *in vivo* counterpart. **(E)** Venn diagram distribution of common persistent *iv-*expanded clones between PRE, POST-2, and POST-3 samples (expansion ≥ 10%). **(F)** Cumulative frequency distribution of common *iv-*expanded clones between the different samples. **(G)** N° and cumulative frequency distribution of *iv-*expanded clones in VAC-SITE/C-MTS compartments. **(H)** Distribution of *in vivo* MIFC regarding their *in vitro* frequency-expansion (iv+, iv–) and their initial abundance (pre-existing basal clones; PRE > 0, and very-low-frequency clones, VLF), in VAC-SITE/C-MTS compartments. **(I–K)** Paired-specific redundancy of *iv-*expanded clones relative to their *in vivo* counterpart. *Samples*: PRE = P0 = PBMC-PRE; POST-2 = P2 = PBMC-POST-2; POST-3 = P3 = PBMC-POST-3. IV; *iv*: *in vitro*. All Wilcoxon test, ****p < 0.0001.

Recognition of vaccine Ags by pt-045 lymphocytes increased markedly during CSF-470 immunization as measured by IFN-γ ELISPOT (Figure 4A). A memory response from childhood vaccination was evident in all PBMC samples stimulated with BCG (*not shown*). It was of essential interest to characterize the TCRβ repertoire after *in vitro* stimulation of PBMC with vaccine-lysate to determine if certain clonotypes were preferentially stimulated. Therefore, we exposed PBMC samples to CSF-470 vaccine-lysate under the same conditions as for ELISPOT assay and performed TCRβ sequencing analysis (Supplementary Tables 6–8, Supplementary Material 1, Supplementary Figure 8A). We found that clonotypes present in VAC-SITE/C-MTS tissue repertoire presented increased frequency distribution after *in vitro* stimulation as compared to non-tissue related clones (Supplementary Figures 8B–D). In particular, frequency of persistent clones (those found in POST-3 sample) was also increased relative to non-persistent clones after stimulation (Supplementary Figures 8E–G); thus further analysis was focused on this subset. Persistent TCRβ clones expanded after *in vitro* stimulation relative to their *in vivo* counterparts in all PBMC samples as seen in paired graphs (expansion ≥10%) (Figures 4B–D). Distribution of common persistent- *in vitro****-*** expanded clones of PRE, POST-2 and POST-3 blood samples revealed a core of common clones that expanded the most for all samples (*n* = 86) (Figure 4E). This core of clones accounted for 65% of expanded clones in PRE-sample and about 40–50% in post-vaccination samples (Figure 4F). Other clonotypes, not found in PRE-sample, were expanded in POST-2 and POST-3 *in vitro* stimulated PBMC, suggesting that additional clonotypes were stimulated after vaccination (Figure 4F). In all samples, clonotypes from all VAC-SITE/C-MTS compartments were expanded; the more abundant clonotypes (higher cumulative frequency) were those present both at the VAC-SITE and C-MTS (Figure 4G). For the *in vivo* TCRβ repertoire (just as obtained from the patient), we have previously defined a subset of clonotypes that markedly increased their frequency after vaccination (MIFC) as compared to PRE sample (Figures 3B–D). Tracking of these MIFC among all *in vitro*-expanded clones revealed that 30% of them were expanded *in vitro* in response to vaccine-Ags (Figure 4H). Mainly, VLF clones were expanded in this experiment.

Finally, paired-specific redundancy of *in vitro*-stimulated clones was increased relative to their *in vivo* counterparts (Figures 4I–K); 27% of *in vivo* MIRC were expanded *in vitro* (*not shown*).

## Discussion

In this study, we examined a metabolically-active chronic VAC-SITE and correlated its lymphoid infiltrate with that present in a C-MTS that appeared during immunization. We also analyzed the clonal TCRβ dynamics in blood throughout CSF-470 immunization protocol. Since BCG was an adjuvant in our CSF-470 cellular vaccine, we asked if the strong BCG immunogenicity was detrimental to the immune response toward tumor Ags. Previous work from our group suggested that this was not the case, since a potent Th1 response toward vaccine-Ags was obtained (14, 17). In pt-045, vaccination induced chronic granulomas resembling chronic *M. tuberculosis* granulomas (18, 19), with a central, necrotic, and caseous core surrounded by a lymphoid structure. A highly-organized structure of clusters of CD11c^+^ cells co-expressing PDL-1 was observed, suggesting a role in local Ag presentation. CD8^+^ lymphocytes and CD68^+^ macrophages were abundant and intersparsed among CD11c^+^ clusters, whereas PD1^+^ and FOXP3^+^ lymphocytes were scarce. The maturity of the granuloma was corroborated by the presence of characteristic LMGC, devoid of BCG bacilli. Notably, in a previous, acute CSF-470 VAC-SITE which developed after only one immunization, LMGC were scarce and only had few nuclei (20). Although the function of LMGC remains to be defined, *in vitro* experiments suggest that they arise after interaction between bacterial lipomannan and macrophages (21). Lay et al. showed in an experimental granuloma system that LMGC with a large number of nuclei were unable to phagocytose *M. tuberculosis* but retained Ag-presentation capacity (22), and Byrd proposed that LMGC induced by IFN-γ/IL-3 are a hallmark of an effective immune response in mycobacterial infection (23). Previous *in vitro* experiments demonstrated that BCG-induced strong-release by monocytes of the pro-inflammatory cytokines TNF-α and Il-1β, and that such release was tempered by the presence of vaccine irradiated-tumor cells (24). We suggest that the final granuloma structure at VAC-SITE is shaped by the interaction among BCG, irradiated CSF-470 cells and incoming immune cells.

Although blood CD4^+^ and CD8^+^ T lymphocyte numbers did not increase in periphery throughout vaccination (14), we observed brisk-lymphocyte infiltration in the C-MTS, with a markedly increased relative to the primary tumor, confirming a previous observation (16). Such increase in C-MTS TIL is important, since tissue microarrays performed on excisional biopsies from 95 patients revealed that brain and skin metastases were less infiltrated by TIL than other sites (25), and coincides with another analysis of 192 CM metastases, in which 159 patients lacked an inflammatory component and only 33 patients presented a mild-to-moderate inflammatory component (26). It has been reported that TILs in melanoma have a positive prognostic value of a relevant immune response toward tumor cells (27). Also, TILs have been shown to be reactive against shared tumor antigens and neoantigens (28). Besides, increases in the DTH-score and T-cell reactivity against vaccine Ags by ELISPOT during vaccination was observed for most CSF-470 vaccinated patients (14).

Analysis of the CDR3-TCRβ is commonly used as a molecular barcode as it allows analyzing the distribution of T-cell clones among the different body compartments and their dynamics. However, additional diversity is achieved by different combinations of α and β chains giving rise to the full functional TCR, with the potential to cover the broad repertoire of antigens (29). We analyzed if the T-cell repertoire present at the VAC-SITE was related to that infiltrating the C-MTS. Indeed, 37% of C-MTS TIL TCRβ clonotypes were common to those found at the VAC-SITE and had expanded in frequency. The majority of shared clones persisted in blood by the end of the CASVAC-0401 protocol (PBMC-POST-3), suggesting the induction and persistence of a specific immune response. Such persistent clonotypes remained at high frequency in blood; most-frequent clonotypes are usually related to a relevant biological role (30). Persistent MIFC were also detected in both VAC-SITE/C-MTS compartments, suggesting the expansion of clonotypes recognizing common as well as private Ags. Importantly, increased MIFC expansion was detected for common VAC-SITE/C-MTS clones derived from peripheral basal pre-existing clones, suggesting that such clonotypes might recognize shared melanocyte-differentiation Ags and cancer-testis Ags, known to be expressed by the CSF-470 vaccine (14).

*In vitro* stimulation of PBMC obtained before (PRE) and after vaccination with CSF-470 lysate expanded 30% of persistent MIFC found at VAC-SITE/C-MTS. Persistent clones previously found to be shared only with C-MTS were also expanded, suggesting that vaccine Ags induced a broader TCRβ repertoire than that detected in the VAC-SITE. Other persistent MIFC expanded *in vivo* were not expanded *in vitro*; these clonotypes might be recognizing private Ags present not in the vaccine. In recent work, TCRβ sequencing was used to address the origin of clinically relevant T-cell clones following adoptive cell therapy, and it was found that VLF clones expanded and persisted in time in those patients who achieved a complete response (31). Accordingly, among persistent clones we found that 75% of MIFC were also VLF clones which emerged during immunization.

Long-term persistence of vaccine-induced T-cell repertoires was shown by TCR clonotyping and multimer staining in other vaccination systems. Evidence was shown for peptide-based vaccines with MART-1 (32, 33) and MAGE (34) Ags for melanoma; with cancer-testis Ag for lung cancer (35); with glypican-3 Ag for hepatocellular carcinoma (36), and with allogeneic cell-vaccines combined with ICKB for pancreatic ductal adenocarcinoma (37). Finally, persistent TCR repertoires were shown in dendritic cell-based personalized vaccine targeting neoantigens and gp100 peptides (38), and in TRP2 mRNA-electroporated Langerhans cells in phase I trials for melanoma (39).

Our results from pt-045 showed that CSF-470 immunization induced the expansion and emergence of a T-cell repertoire able to infiltrate tumor sites, as described previously for pt-006 (16); with analysis for the first time of VAC-SITE clonotypes. That new clonotypes still appeared by the end of the immunization protocol suggests that repeated prolonged vaccination is useful. Interestingly, when analyzing the distribution of redundant clonotypes in all samples, we found that VAC-SITE and C-MTS presented high specific redundancy, with even >20 TCRβ clones with different nucleotide sequences converging to the same aminoacidic sequence. This might have functional implications at the VAC-SITE, where Ag-stimulation is being seeked, as well as in the C-MTS, where effective T-cell effector function is desired. These results support the hypothesis that biologically relevant T-cell clones might expand through the proliferation of several redundant TCRβ clones, underlying a functional antigenic selection. Noticeably, the proportion of redundant clones in PBMC-POST-3 sample was preferentially distributed into those shared either with the VAC-SITE and/or the C-MTS. Clonotypes also exhibited increased specific redundancy when stimulated *in vitro* with the vaccine lysate. Accordingly, all subsets of persistent clones presented markedly-increased redundant clones, prevailing expansion of those MIRC in common with the VAC-SITE/C-MTS. Therefore, either emergence or expansion of persistent clones induced throughout CSF-470 immunization might stem from the proliferation of clones as well as from an increase in their specific redundancy. It is our goal in the near future to perform NGS sequencing of this patient's tumor to predict neo-Ags and to test PBMC reactivity both to common and private peptide-Ags throughout the CSF-470 vaccine immunization protocol. It would be most interesting to analyze TCR repertoires at the antigen-specific level, tracking their distribution at the VAC-SITE and the C-MTS as well as their changes in frequency and redundancy in comparison to the present work.

## Conclusion

In this case report of pt-045 from CASVAC-0401 protocol, we found that immunization with the CSF-470 vaccine plus BCG and rhGM-CSF induced an immune repertoire of T lymphocytes at the VAC-SITE, with a related increased immune infiltration in a regional C-MTS, resulting in the expansion of Ag-boosted T-cell clones which persisted in blood by the end of the 2-yr vaccination.

## Data Availability

The datasets generated for this study can be found in ImmuneACCESS, Adaptive Biotechnologies, and are provided in Supplementary Tables 1–8.

## Ethics Statement

The CASVAC-0401 study was carried out in accordance with the recommendations of the Ethics Committee of the Instituto Alexander Fleming, with written informed consent from all subjects. All subjects gave written informed consent in agreement with the Declaration of Helsinki. Pt-045 specifically consented to publish the results obtained referring to his participation in the clinical study CASVAC-0401, providing anonymity was assured. The protocol was approved by the Prof. Luis María Zieher Independent Ethics Committee for Clinical assays in Clinical Pharmacology (Argentina), and the Ethics Committee of the Instituto Alexander Fleming (Buenos Aires, Argentina), and by the Argentine Regulatory Agency (ANMAT) (Disposition 1299/09). The Ethics Committee of the Instituto Alexander Fleming (Buenos Aires, Argentina) is reputed by the Central Ethics Committee of the City of Buenos Aires (Argentina).

## Author Contributions

MA, MB, and JM: conception and design, collection and assembly of data, data analysis and interpretation, and manuscript writing. MB and JM: Sub/Principal Investigator of the CASVAC-0401 study. HG, IC, and MN: data analysis and interpretation, and manuscript writing. AB, EP, PB, CR, and SB: collection and assembly of data.

### Conflict of Interest Statement

The authors declare that the research was conducted in the absence of any commercial or financial relationships that could be construed as a potential conflict of interest.

## Supplementary Material

Supplementary Table 1 PBMC-PRE productive rearrangements.

Supplementary Table 2 PBMC-POST-2 productive rearrangements.

Supplementary Table 3 PBMC-POST-3 productive rearrangements.

Supplementary Table 4 VAC-SITE productive rearrangements.

Supplementary Table 5 C-MTS productive rearrangements.

Supplementary Table 6 PBMC-PRE-IV productive rearrangements.

Supplementary Table 7 PBMC-POST-2-IV productive rearrangements.

Supplementary Table 8 PBMC-POST-3-IV productive rearrangements.

## Abbreviations

ABC: Avidin-Biotin-Peroxidase
Ags: antigens
BCG: Bacillus Calmette Guerin
CM: cutaneous melanoma
C-MTS: cutaneous metastasis
DTH: Delayed Type Hypersensitivity
FFPE: Formalin-fixed, paraffin-embedded
GZMB: granzyme B
HE: hematoxylin-eosin
HLA: human leucocyte antigen
ICKI: immune checkpoint inhibitors
IFN-α2b: interferon alpha 2b
IFN-γ: interferon-gamma
LMGC: Langhans multinucleated giant cells
LN: lymph node
MAbs: monoclonal antibodies
MIFC: markedly-increased frequency clones
MIRC: markedly-increased redundant clones
PHA: Phytohemagglutinin-A
PBMC: Peripheral blood mononuclear cells
Pt: patient
rhGM-CSF: recombinant human granulocyte macrophage-colony stimulating factor
SLNB: sentinel lymph node biopsy
TCRβ: T-cell receptor beta
TIL: tumor infiltrating lymphocytes
VAC-SITE: vaccination site
VLF: very low frequency.
