# Supplementary material for: Immunization With the CSF-470 Vaccine Plus BCG and rhGM-CSF Induced in a Cutaneous Melanoma Patient a TCRβ Repertoire Found at Vaccination Site and Tumor Infiltrating Lymphocytes That Persisted in Blood
Source: Front Immunol. 2019 Sep 18;10:2213. doi: 10.3389/fimmu.2019.02213 (PMC6759869; doi:10.3389/fimmu.2019.02213)
Supplement: Supplementary file 9 [file Data_Sheet_1.pdf]

SUPPLEMENTARY MATERIAL

MATERIALS AND METHODS

Patients

Pt-045 is a 51 year-old man, to whom in 12/2015 a dorsal melanoma with a 2.3 mm Breslow index was excised. A micrometastasis in a sentinel node at the right axilla was detected, but axillary dissection was not performed at the time. The patient was therefore at stage III of the disease, and he entered the CASVAC 0401 study on 04/21/2016. After signing informed consent, he was randomized to the vaccine arm. After receiving 8 vaccinations, on 05/2017, he progressed to a lymph node adenopathy and a dorsal subcutaneous nodule (C-MTS) which were both excised. Pathology revealed metastasis at both locations. C-MTS expressed MD-Ag MART-1 and gp100 as determined by immunohistochemistry (*not shown*). Simultaneously, several vaccination sites detected by a PET-CAT scan were also excised. Since both metastases were loco-regional, the patient remained in the study as permitted by protocol, and at 11/2018 he is disease-free 36 months after initial surgery.

PBMC samples, haplotype, and immune cell population analysis from pt-045

During CASVAC-0401 protocol, peripheral blood samples were obtained from this patient at 0 months (PRE), 6 months (POST-1), 18 months (POST-2) and 25 months (POST-3) after starting immunization with CSF-470 vaccine (Mordoh, 2018). Peripheral blood mononuclear cells (PBMC) were obtained by density gradient purification and cryopreserved at N_2. POST-1 sample was not analyzed due to low quality of cell recovery. HLA haplotype was determined from PBMC by Scisco Genetics (Nelson et al.); presenting haplotype A*29:02:01 A*29:02:01; B*44:03:01 B*44:03:01; C*16:01:01 C*16:01:01; DPA1*01:03:01 DPA1*02:01:01; DPB1*04:01:01 DPB1*11:01:01; DQA1*02:01:01 DQA1*05:05:01; DQB1*02:02:01; DQB1*03:01:01; DRB1*07:01:01; DRB1*11:01:01;DRB3*02:02:01 DRB4*01:01:01:01.

Histopathological and immunohistochemical analysis of biopsies

Histopathological features were determined in formalin fixed paraffin embedded samples (FFPE) according to AJCC-UICC staging (Balch, 2009). Proliferative index was determined by Ki-67+ staining (4) (PI) (%): Ki-67^+tumor cells/ (Ki-67^+ tumor cells + Ki-67^-tumor cells) x100. Biopsies were stained with the following anti-

human MAbs: CD8 (C8/144), CD20 (L26), CD45Ro (UCHL1) and CD68 (PG-M1) from Dako, Denmark; Foxp3 (236A/E7), CD11c (EP1347Y), GZMB (EPR8260) and PD-1 (NAT105) from Abcam, MA, USA; CD4 (1F6) from Novocastra, Wetzlar, Germany; HLA-I (EMR8-5) from Abcam and PD-L1 (E1L3N) from Cell-Signalling Technology, MA, USA. The Avidin-Biotin-Peroxidase (ABC) system (Vectastain, Vector Labs) was used. Sections were examined by optical microscopy (Olympus BX40 microscope, DP2-BSW software). To analyze VAC-SITE and C-MTS, slides images were captured using a RGB video camera (Olympus DP73, Japan) attached to a wide-field transmitted light microscope (Olympus BX53, Japan) equipped with a motorized platina (PRIOR H101AFI, USA). Images were captured using a 20x objective. For capturing the entire area, the Multiple Image Alignment option of the CellSens Dimension v1.7 image analysis software (Olympus, Japan) was applied (Facultad de Ciencias Veterinarias Universidad Nacional de La Plata, Argentina).

Delayed-Type Hypersensitivity (DTH)

On the vaccination day, DTH was performed in the forearm with 1/10th of the CSF-470 dose. The reaction was measured at 1, 24, 48, and 72 h and recorded as follows: 0: macular erythema < 0.5 cm diameter; 1: macular erythema 0.5–1.0 cm; 2: macular erythema 1.1–2.0 cm; 3: macular erythema > 2.0 cm; and 4: papular erythema > 2.0 cm. A DTH score corresponding to the sum of the four values was calculated for each vaccination.

***In vitro* stimulation by CSF-470 vaccine-lysate / ELISPOT**

For IFN-gamma ELISPOT assay, PBMC (PRE, POST2 and POST3) cells were thawed and then seeded (1×10^6) in 1 mL of Complete Medium consisting of RPMI 1640 (Invitrogen, USA) supplemented with 10% heat-inactivated human AB sera, 2 mM glutamine, 100 U/mL penicillin, 100µg/mL streptomycin, 2.5 mM HEPES and 50 U/mL of IL-2 (Laboratorio Pablo Cassará SRL, Argentina), in 24-well plates (Costar, Corning, USA). PBMC were stimulated with CSF-470 lysate in a 3:1 ratio (Ag-presenting cells from PBMC population: lysed CSF-470 cells), or sonicated BCG (20µg/ml, Pasteur strain, (Instituto Malbrán, Argentina)) and cultured at 37◦C, in 5% CO2 for 12 days, as described. ELISPOT assay was performed as described (Pampena, 2018). 275.000 effector cells were plated per well. Plates were scanned using an AID iSPOT ELR088IFL analyzer

and AID Elispot reader software 7.0 (AID, Germany) was used to quantify the number of spots per well. Spots could be quantified appropriately from 1 to 350 spots/well due to saturation of the signal. The positive controls consisted of the OKT-3 and PHA re-stimulated wells; subtracted background signal (negative control) was obtained from wells containing unstimulated PBMC plus culture medium.

TCRβ immune repertoire analysis

Genomic DNA was isolated from total PBMC samples (PBMC-PRE, PBMC-POST-2, PBMC-POST-3), *in vivo* and *in vitro* stimulated counterparts, using Quick-DNA (Zymo); and from tissue samples (VAC-SITE & C-MTS) with RecoverAll (Ambion) kits. DNA was verified by spectrophotometry (Nanodrop). VAC-SITE and C-MTS immune infiltrated areas were purified by laser microdissection (Leica DM/LAM). High-throughput next-generation sequencing of the T-cell receptor beta (TCRβ) CDR3 region was performed through the Immunoseq platform, at deep-resolution for PBMC samples and survey-resolution for tissue samples (Adaptive Biotechnologies, USA). This technology is based on multiplex PCR, amplifying at the DNA level from T lymphocytes all VJ-gene combinations from the CDR3 region of the TCR β chain, followed by NGS sequencing. It combines the use of a synthetic repertoire with all VDJ gene combinations along with bioinformatics error correction methods in order to optimize multiplex PCR reactions to correct for bias amplification and quantify the different clonotypes in each sample. Each survey-level sample is performed with two replicates reactions (VAC-SITE & C-MTS samples) and each deep-level sample is performed with five to six replicate-level replicate reactions (PBMC-PRE, POST-2 & POST-3).

Sequences are available at **Supplementary tables 1-8**. For analysis were considered productive amino-acid rearrangements, in-frame unique sequences without stop codons, with starting and ending positions matching reference V and J genes sequences, resulting in C or Y (depending on the corresponding V gen) as initial position and F as final position. To compare datasets with different size, the bootstrapping method was applied to them to calculate the mean and errors for 1000 iterations (Supplementary Figure 4E, n=1000; Figure 3C, n=300).

TOP100 clones are the 100 most-frequent clones, ordered by frequency. *Redundant clones* were defined as TCRβ clonotypes formed by more than one rearrangement. *Specific redundancy* was defined as the number of

rearrangements/redundant clone. *Persistent clones* were defined as those clones present at PBMC-POST-3 sample that were also present either at the VAC-SITE or the C-MTS. *Marked-increased frequency clones (MIFC)* were defined as persistent clones which at least doubled their frequency throughout immunization (). *Marked-increased redundant clones (MIRC)* were defined as persistent clones which at least doubled their specific redundancy throughout immunization. *Very-low frequency clones (VLF)* were defined as those where frequency at PBMC-PRE proceeded from zero/one/two templates; while *basal pre-existing clones* contained at least 3 templates. Analysis of *in-vitro* expanding clones included those with an expansion of at least 10%. Wilcoxon test was applied, $p<0.05$ indicated statistically significant differences.

Subsets at Venn diagram: P03∩VS∩CM, shared clonotypes among POST-3, VAC-SITE and CM samples; P03∩VS∩CM´, shared clonotypes among POST-3 and VAC-SITE samples; P03∩VS∩CM´, shared clonotypes among POST-3 and CM samples; P03∩VS´∩CM´, clonotypes found only at POST-3 sample; VS∩CM∩P03´, shared clonotypes among VAC-SITE and C-MTS samples; VS∩P03´∩CM´, clonotypes found only at VS sample; CM∩ P03´∩VS´, clonotypes found only at CM sample.

Blood TCRβ clone-tracking patterns throughout CSF-470 immunization were defined using a K-means clustering method from Scikit-learn version 0.20.2 Python library with K=9 (number of clusters). Before running the clustering algorithm, clone frequencies were normalized to the maximum frequency registered in the time series (PBMC-PRE=0, PBMC-POST-2=2 & PBMC-POST-3=3). Only clones with at least 3 templates in one PBMC sample/time point were considered for this analysis (n=7975). Clones present in two or more samples/time points were grouped applying the clustering method while clones present in only one sample were grouped separately (patterns 4, 8 and 12). In order to study the pattern distribution of persistent clones relative to non-persistent ones (baseline), the log odds-ratio was calculated for each pattern within each of the 3 subsets of persistent clones (P03∩VS∩CM, P03∩VS´∩CM & P03∩VS∩CM´). For instance, the log odds-ratio for pattern 1 in subset P03∩VS∩CM was calculated applying the log to the ratio of the proportion of clones in pattern 1 within the subset P03∩VS∩CM to the proportion of clones in pattern 1 within the subset P03∩VS´∩CM´.
