# Supplementary material for: Immunization With the CSF-470 Vaccine Plus BCG and rhGM-CSF Induced in a Cutaneous Melanoma Patient a TCRβ Repertoire Found at Vaccination Site and Tumor Infiltrating Lymphocytes That Persisted in Blood
Source: Front Immunol. 2019 Sep 18;10:2213. doi: 10.3389/fimmu.2019.02213 (PMC6759869; doi:10.3389/fimmu.2019.02213)
Supplement: Supplementary file 10 [file Image_1.pdf]

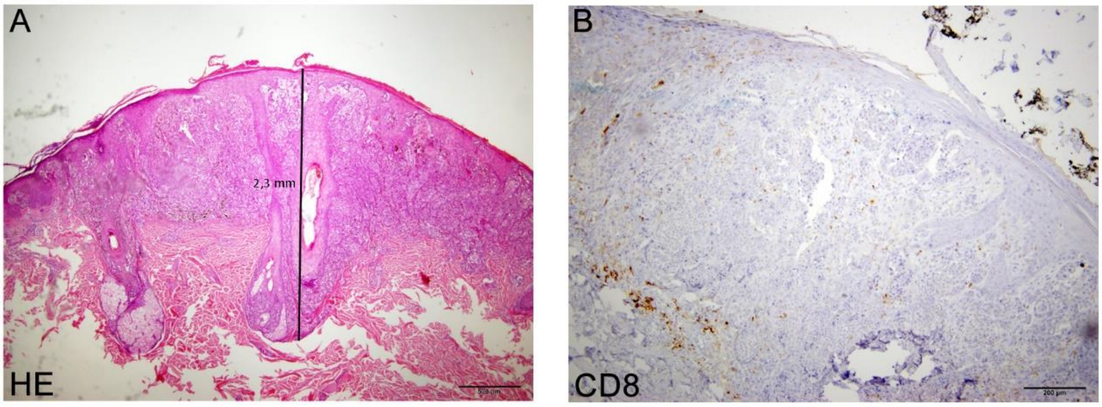

Supplementary Figure 1. Histology of pt-045 primary tumor. (A) HE staining of the cutaneous melanoma; Breslow thickness is indicated. (B) Immunohistochemistry revealed scarce $CD8^+$ lymphocytes infiltrating the tumor. Original magnifications: 40X (A), 100X (B). Scale bars: 500 µm (A), 200 µm (B).
