# Supplementary material for: Immunization With the CSF-470 Vaccine Plus BCG and rhGM-CSF Induced in a Cutaneous Melanoma Patient a TCRβ Repertoire Found at Vaccination Site and Tumor Infiltrating Lymphocytes That Persisted in Blood
Source: Front Immunol. 2019 Sep 18;10:2213. doi: 10.3389/fimmu.2019.02213 (PMC6759869; doi:10.3389/fimmu.2019.02213)
Supplement: Supplementary file 11 [file Image_2.pdf]

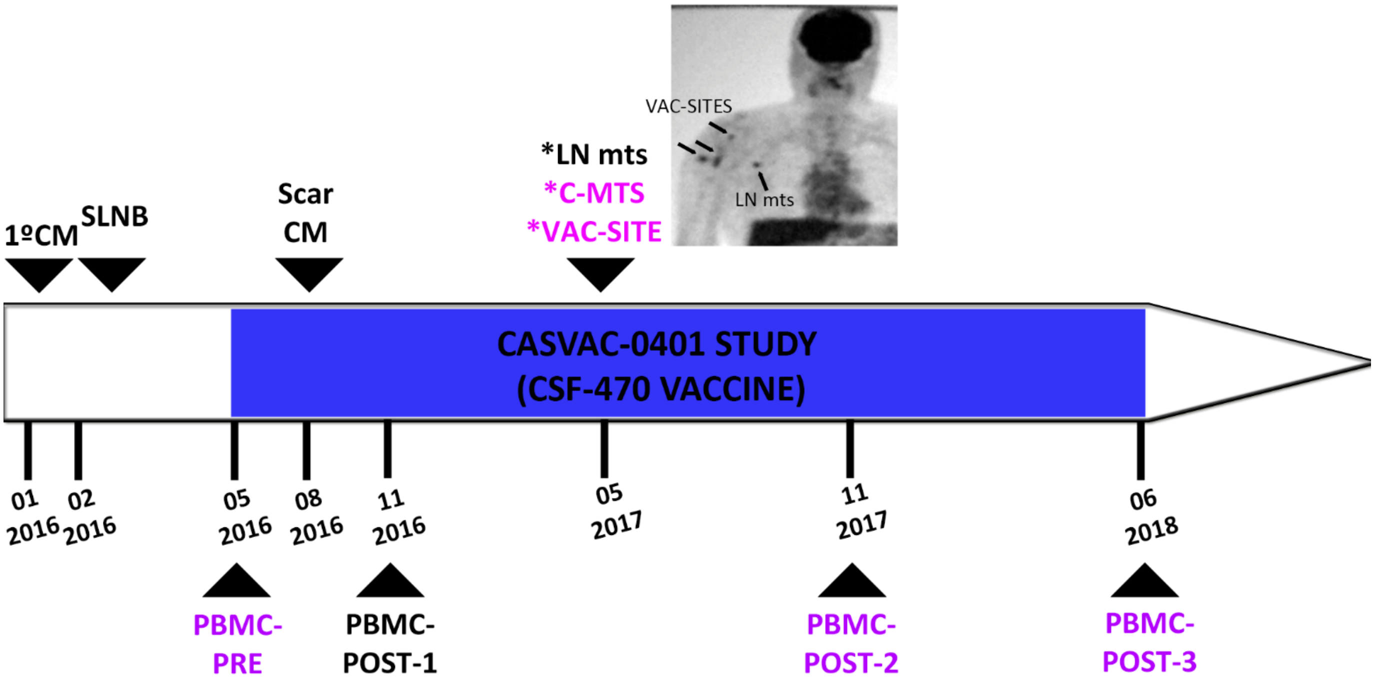

Supplementary Figure 2. Pt-045 timeline. (A) The pt-045 was randomized to the CSF-470 vaccine arm of the CASVAC-0401 study. Tumor resections of primary cutaneous melanoma (1ºCM), sentinel lymph node biopsy (SLNB), Scar-CM, cutaneous metastasis (C-MTS) and inoculation sites (VAC-SITE) are indicated by triangles. Peripheral blood mononuclear cell (PBMC) samples were obtained at 0 (PBMC-PRE), 6 (PBMC-POST-1), 18 (PBMC-POST-2) and 25 months (PBMC-POST-3). Samples selected for TCRβ sequencing are shown in violet. *Inset:* LN-MTS and VAC-SITES detected by PET are indicated with arrows.
