# Supplementary material for: Immunization With the CSF-470 Vaccine Plus BCG and rhGM-CSF Induced in a Cutaneous Melanoma Patient a TCRβ Repertoire Found at Vaccination Site and Tumor Infiltrating Lymphocytes That Persisted in Blood
Source: Front Immunol. 2019 Sep 18;10:2213. doi: 10.3389/fimmu.2019.02213 (PMC6759869; doi:10.3389/fimmu.2019.02213)
Supplement: Supplementary file 12 [file Image_3.pdf]

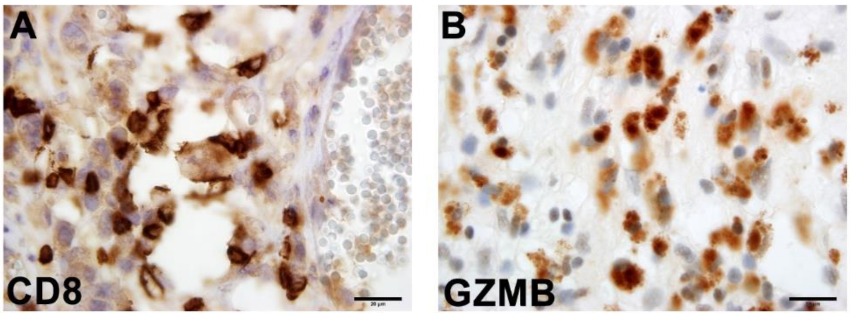

Supplementary Figure 3. *In-situ* tumor cell lysis. In areas of $CD8^+$ lymphocytes infiltration (A), tumor cells were full of granzyme B^+ granules (B), many of them in the nuclei. Original magnification: 1000X. Scale bars= 20µm.
