# Supplementary material for: Immunization With the CSF-470 Vaccine Plus BCG and rhGM-CSF Induced in a Cutaneous Melanoma Patient a TCRβ Repertoire Found at Vaccination Site and Tumor Infiltrating Lymphocytes That Persisted in Blood
Source: Front Immunol. 2019 Sep 18;10:2213. doi: 10.3389/fimmu.2019.02213 (PMC6759869; doi:10.3389/fimmu.2019.02213)
Supplement: Supplementary file 13 [file Image_4.pdf]

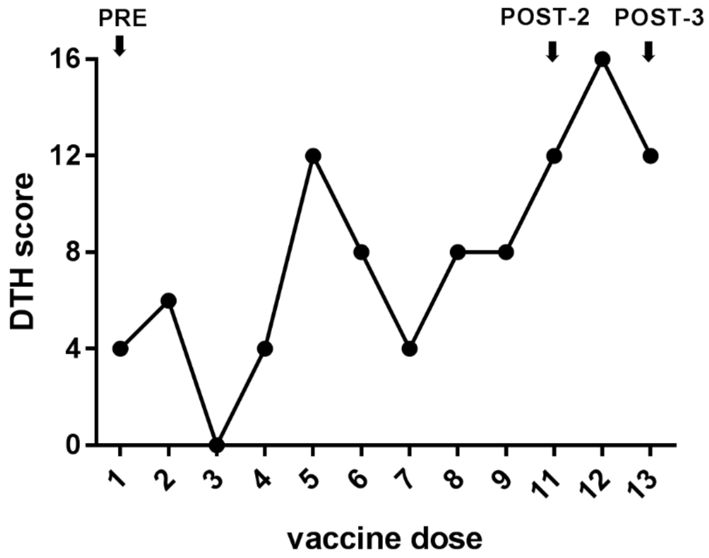

Supplementary Figure 4. Analysis of DTH response following CSF-470 immunization. A skin DTH-score obtained for pt-045 at each of the 13 vaccinations during CASVAC-0401 protocol are shown; vaccine dose number 9 was skipped due to surgery of the C-MTS. PBMC samples obtained before (PRE), at 18 and 25 months respectively (POST-2 and POST-3), are indicated with arrows.
