# Supplementary material for: Immunization With the CSF-470 Vaccine Plus BCG and rhGM-CSF Induced in a Cutaneous Melanoma Patient a TCRβ Repertoire Found at Vaccination Site and Tumor Infiltrating Lymphocytes That Persisted in Blood
Source: Front Immunol. 2019 Sep 18;10:2213. doi: 10.3389/fimmu.2019.02213 (PMC6759869; doi:10.3389/fimmu.2019.02213)
Supplement: Supplementary file 14 [file Image_5.pdf]

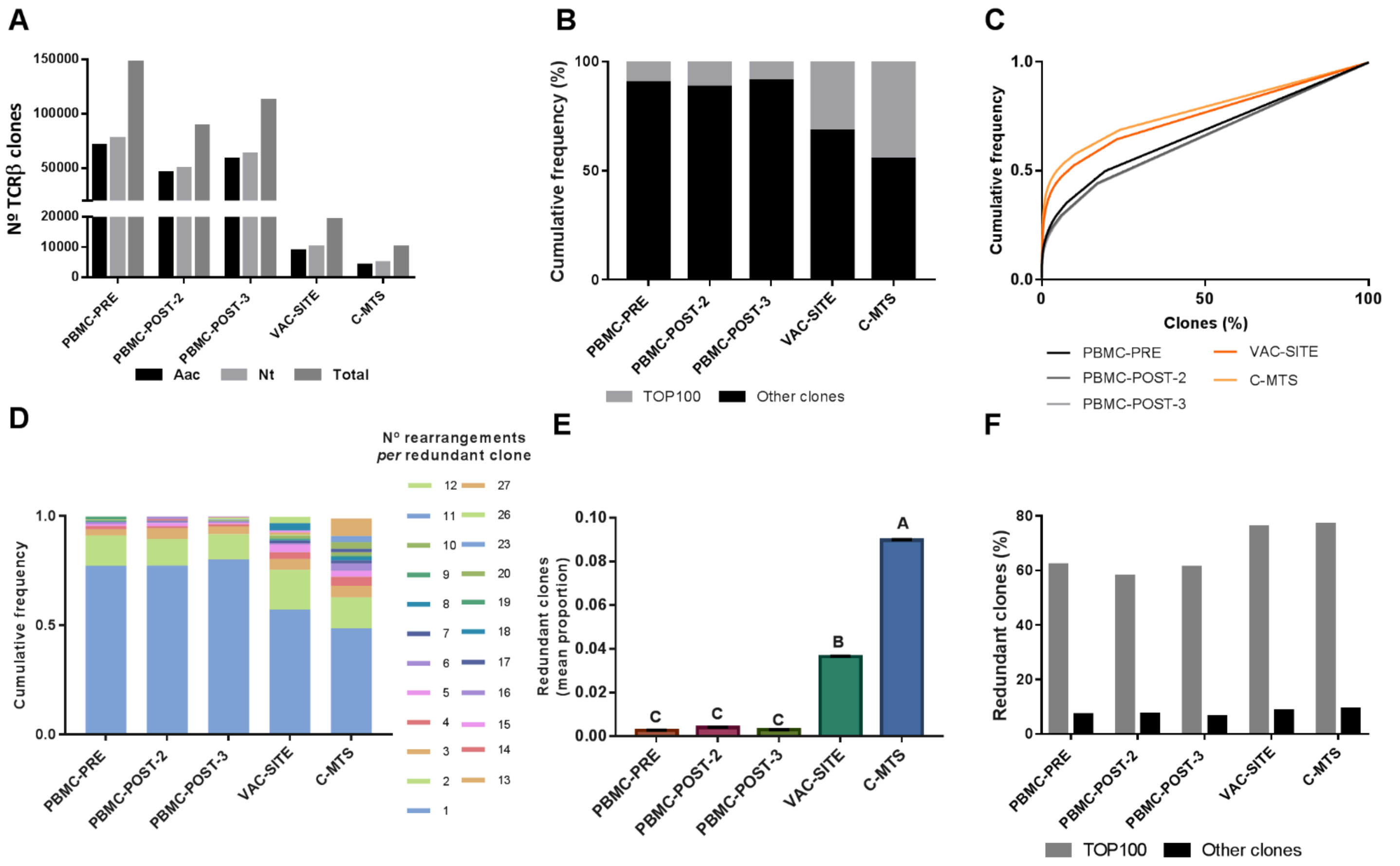

Supplementary Figure 5. Metrics of the TCRβ immune repertoire in PBMC, VAC-SITE and C-MTS. (A) Number of unique amino-acidic (aa) and nucleotide sequences (nt) TCRβ clonotyes, as well as total TCRβ clonotypes analyzed for each sample. (B) Cumulative frequencies by the TOP100 clones for each sample, defined as the 100 most frequent ones. (C) Frequency distribution of TCRβ clones, ordered from highest to lowest frequency, were plotted as function of their cumulative frequencies. (D) Distribution of the number of redundant clones in function of their cumulative frequencies. (E) Mean proportion of redundant clones (bootstrap n=1000, 100 iterations); bars with different letters are statistically different ($p<0.05$). (F) Proportion of redundant clones in TOP100 and other clones. *Samples:* PBMC-PRE, PBMC-POST-2, PBMC-POST-3, VAC-SITE and C-MTS.
