# Supplementary material for: Immunization With the CSF-470 Vaccine Plus BCG and rhGM-CSF Induced in a Cutaneous Melanoma Patient a TCRβ Repertoire Found at Vaccination Site and Tumor Infiltrating Lymphocytes That Persisted in Blood
Source: Front Immunol. 2019 Sep 18;10:2213. doi: 10.3389/fimmu.2019.02213 (PMC6759869; doi:10.3389/fimmu.2019.02213)
Supplement: Supplementary file 15 [file Image_6.pdf]

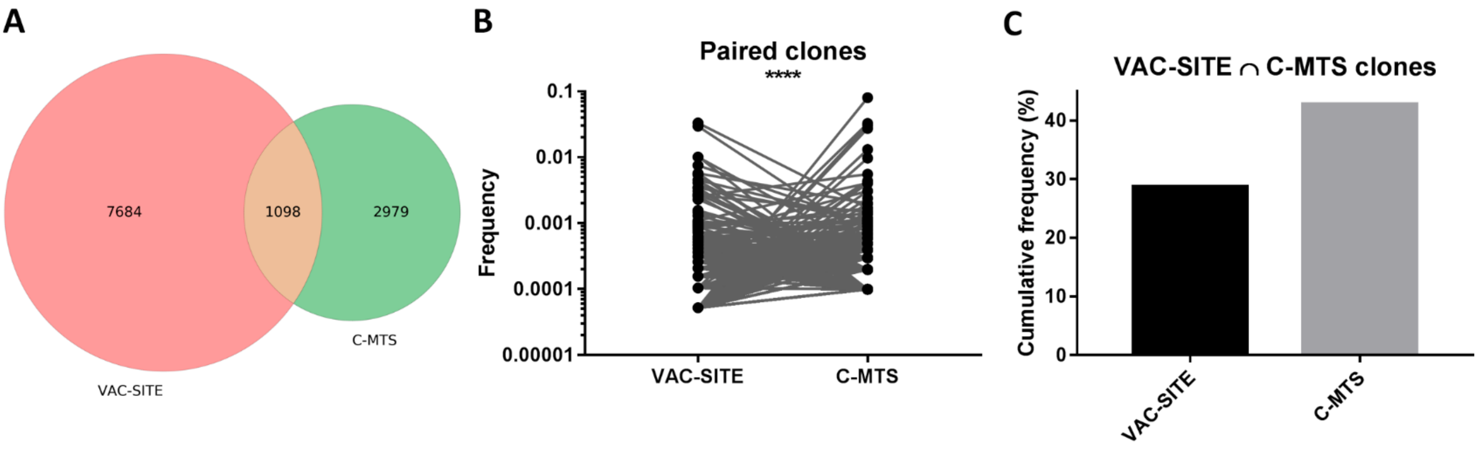

Supplementary Figure 6. Common clonotypes co-exist in a CSF-470 VAC-SITE and C-MTS. (A) Shared TCRβ clonotypes between the VAC-SITE and the C-MTS; (B) changes in the frequency of paired clones (Wilcoxon test, **** indicates $p<0.0001$); and (C) cumulative frequencies (%).
