# Supplementary material for: Immunization With the CSF-470 Vaccine Plus BCG and rhGM-CSF Induced in a Cutaneous Melanoma Patient a TCRβ Repertoire Found at Vaccination Site and Tumor Infiltrating Lymphocytes That Persisted in Blood
Source: Front Immunol. 2019 Sep 18;10:2213. doi: 10.3389/fimmu.2019.02213 (PMC6759869; doi:10.3389/fimmu.2019.02213)
Supplement: Supplementary file 16 [file Image_7.pdf]

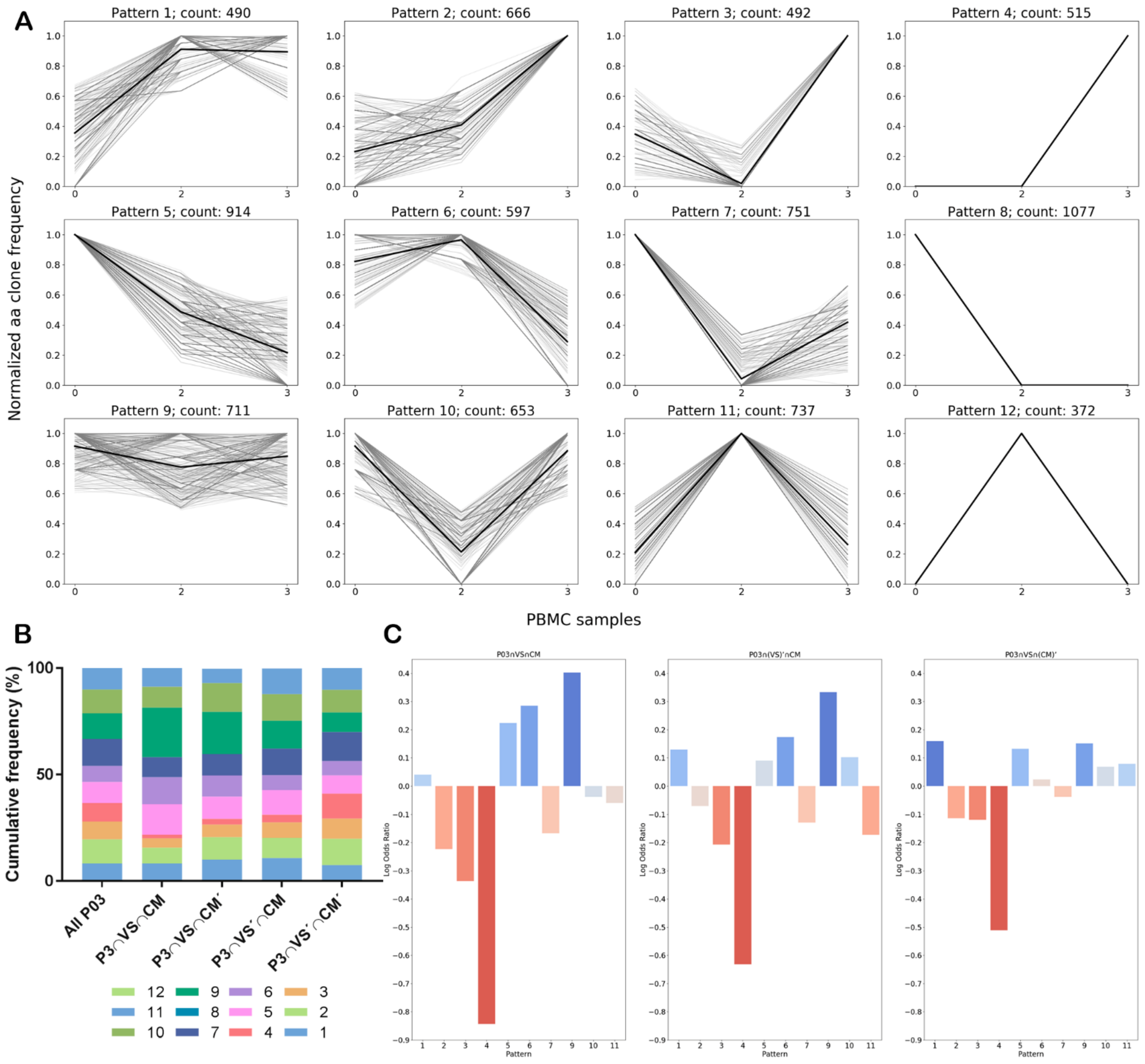

Supplementary Figure 7. Dynamics of TCRβ peripheral repertoire throughout immunization with the CSF-470 vaccine. (A) TCRβ clone-tracking patterns were defined using K-means clustering method (n=7975). (**B**) Cumulative frequency distribution of all POST-3 clones and their different subsets. (**C**) Log odds-ratio for persistent clones at the different patterns relative to non-persistent clones (P3∩VS´∩CM´).
