# Supplementary material for: Immunization With the CSF-470 Vaccine Plus BCG and rhGM-CSF Induced in a Cutaneous Melanoma Patient a TCRβ Repertoire Found at Vaccination Site and Tumor Infiltrating Lymphocytes That Persisted in Blood
Source: Front Immunol. 2019 Sep 18;10:2213. doi: 10.3389/fimmu.2019.02213 (PMC6759869; doi:10.3389/fimmu.2019.02213)
Supplement: Supplementary file 17 [file Image_8.pdf]

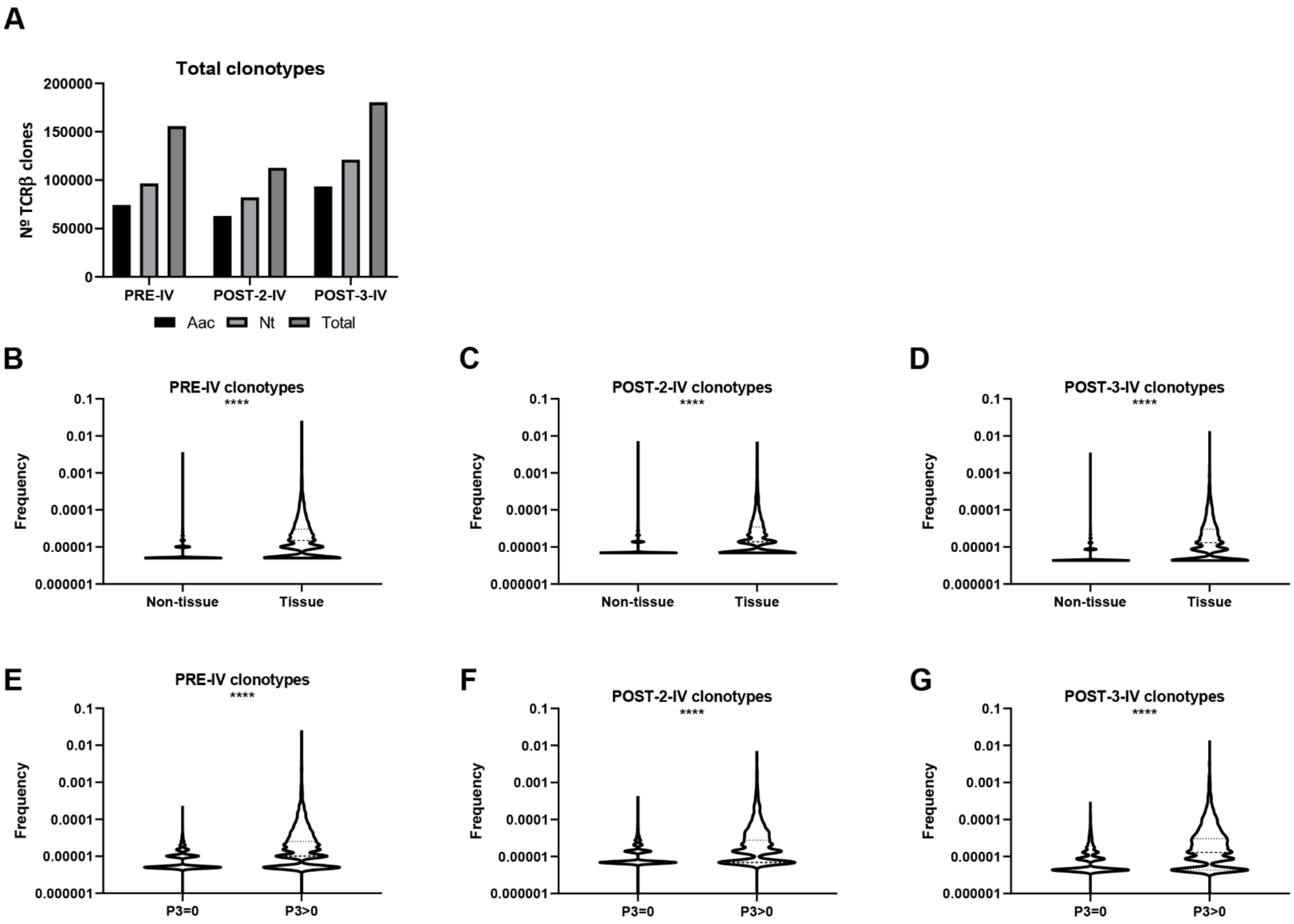

Supplementary Figure 8. Analysis of the TCRβ repertoire stimulated *in-vitro* with CSF-470 vaccine lysate. (A) Number of unique amino-acidic (Aac) and nucleotide sequences (Nt) TCRβ clonotyes, as well as total TCRβ clonotypes analyzed for each sample. **(B-D)** Distribution frequency of TCRβ clonotypes present in tissue (VAC-SITE/C-MTS) and non-tissue from *in-vitro* stimulated PBMC samples: PRE, POST-2, and POST-3. **(E-G)** Distribution frequency of tissue clonotypes in *in-vitro* stimulated samples whether they were persistent (P3>0) or not (P3=0). In all cases, Wilcoxon test was applied; p<0.0001 indicates statistical significance (****).
